# *Culicoides* biting midges among cattle in France: be wary of data in the literature

**DOI:** 10.3389/fvets.2024.1451442

**Published:** 2024-10-24

**Authors:** Christine Millot, Leila Hadj-Henni, Denis Augot

**Affiliations:** ^1^Usc Petard, Anses, EA 7510, SFR Cap Santé, Université de Reims Champagne-Ardenne, Reims Cedex, France; ^2^ANSES, INRAe, ENVA, UMR-BIPAR, Laboratoire de Santé Animale, Maisons-Alfort Cedex, France

**Keywords:** France, cattle, *Culicoides*, vectors, pathogen

## Abstract

*Culicoides* are vectors that can transmit many different pathogens to mammals — including humans, and domestic and wild animals — and birds. In order to take preventive measures against any vector-borne disease, it is important to gather information on both the host and vector species. *Culicoides* species are mainly mammalophilic, ornithophilic or ornithophilic/mammalophilic, but females have also been found to occasionally feed on engorged insects. A recent systematic review based on three groups of key words investigated *Culicoides* on farms, and asserted that 92 species (including four not present species) have been reported among cattle in mainland France and Corsica. We have re-evaluated the presence of *Culicoides* species in cattle in France using the same data of the review. Our data show that only 18 species are reported among cattle. Furthermore, our research used molecular and indirect investigations to analyse *Culicoides* species that had been feeding on cattle. Our results demonstrate that 45 species feed on cattle out of 92 species present in France. The paper discusses the relevance of data in the literature when investigating hosts of *Culicoides* species.

## Introduction

Vector-borne diseases, transmitted by a variety of arthropods, cause health problems for humans, livestock and wild animals. Currently, more than 1,400 *Culicoides* species have been described worldwide (1). *Culicoides* biting midges have been incriminated in the transmission of viruses, protozoa and filarial worms (2). Their economic impact in Europe is due to their transmission of the bluetongue virus (BTV), epizootic haemorrhagic disease virus (EHDV), Schmallenberg virus (SBV) and African horse sickness virus (AHSV). In order to take preventive measures against a vector-borne disease, it is important to gather information on both the host and vector species. Identifying the feeding patterns of *Culicoides* biting midges is an essential step in pathogen circulation in order, for example, to break the transmission chain by vaccinating animals at greatest risk of infection (3).

Generally speaking, *Culicoides* midges have a wide host spectrum and tend to feed on blood opportunistically (4–7); they are classified as mammalophilic, ornithophilic or ornithophilic & mammalophilic species (8–10). *Culicoides* species are more attracted by cattle than sheep (11), and are found in natural areas and on farms. Indeed, the same species feed on both wild and domestic animals, so a switch is possible and could facilitate the circulation of pathogens between these two communities (12). In this context, an accurate knowledge of *Culicoides* species present on farms is essential for anticipating diseases. In France, 92 species of *Culicoides* are reported (13–18). A recent study examining *Culicoides* species on farms in mainland France and Corsica suggested that 94 species (including two invalid species and four not present species) were present among cattle. The study was based on a systematic review using three groups of key words (19). In this context, do these species play an epidemiological role in the transmission of pathogens among cattle? The first step in answering this question is to prove that there is a relationship between the host and the insect. *Culicoides* species-host pairing is a key factor in anticipating and understanding the transmission of vector-borne pathogens. Here, we investigate *Culicoides* species and cattle based on a review in order to research bibliographic references with traditional approach (PCR analysis of blood meals taken from the host by female *Culicoides* in the field) and indirect investigations (animal baits, direct aspiration, light traps and glue strips). We compare the checklist of *Culicoides* species reported in France with their cattle preference. Prudhomme et al. (19) reported that all *Culicoides* species in France are present among cattle and this number of species is superior on other references reported. So, a second objective is to re-evaluate the presence of *Culicoides* spp. in cattle in France.

## Materials and methods

First, in 2012, a list of *Culicoides* present in France was drawn up by Venail et al. (16). The list of *Culicoides* species identified 88 species in all, 83 for mainland France and 61 for Corsica.

Four species have since been added to this preliminary list: (i) *C. paradoxalis* Ramilo and Delécolle, 2013 was identified in mainland France and Corsica (13); (ii) C. *boyi* Nielsen, Kristensen and Pape, 2015 was reported in mainland France (14) on a farm with cattle (20); (iii) *C. bysta* Sarvašová and Mathieu, 2017 was reported in mainland France (15) and (iv) *C. cryptipulicaris* Talavera, Muñoz-Muñoz, Verdún and Pagès, 2017 (13, 17, 18).

The distribution of species is not homogeneous in these four groups: for example Mediterranean littoral and Corsica present the same climate but a species present in Corsica is no necessary present in Mediterranean littoral and *vice-versa* (i.e., *C. corsicus* Kremer, Leberre and Beaucournu-Saguez, *C. derisor* Callot and Kremer, *C. heteroclitus* Kremer and Callot, *C. impunctatus* Goetghebuer, *C. indistinctus* Khalaf, *C. jamaicensis* Edwards, *C. maritimus paucisensillatus* Callot, Kremer and Rioux, *C. minutissimus* (Zetterstedt), *C. montanus* Shakirzjanova, *C. nubeculosus* (Meigen), *C. paradisionensis* Boorman, *C. reconditus* Campbell and Pelham-Clinton, *C. riebi* Delécolle, Mathieu and Baldet, *C. saevus* Kieffer, *C. sahariensis* Kieffer, *C. salinarius* Kieffer, *C. segnis* Campbell and Pelham-Clinton, *C. simulator* Edwards, *C. tauricus* Gutsevich, *C. vexans* (Staeger)). All articles including *Culicoides* spp. and host preference were selected. All publications have been reading. Our study used a traditional approach (PCR analysis of blood meals taken from the host by female *Culicoides*) and indirect investigations (animal baits, direct aspiration, light traps and glue strips) in the Palaeartic region to research the feeding preferences of *Culicoides* spp. The data we found in the literature were from various countries, including the country that first described the type and species in the Palaeartic region (Table 1). The feeding preferences of *Culicoides* biting midge species present in France (Table 1) has been linked to cattle (Table 2).

**Table 1:** Checklist of *Culicoides* species reported in mainland France and Corsica with the countries where types were first identified.

Species name	Country of identification of *Culicoides* type	Distribution	
		Mainland	Corsica
*C. abchazicus* Dzhafaraov, 1964	Georgia	☒	
*C. achrayi* Kettle and Lawson, 1955	Ukraine	☒	☒
*C. alazanicus* Dzhafaraov, 1961	Azerbaijan	☒	☒
*C. albicans* (Winnertz), 1852	Germany	☒	
*C. albihalteratus* Goetghebuer, 1935	France	☒	
*C. begueti* Clastrier, 1957	Algeria	☒	☒
*C. boyi* Nielsen, Kristensen and Pape, 2015	Denmark	☒	
*C. brunnicans* Edwards, 1939	Great Britain	☒	☒
*C. bysta* Sarvašová and Mathieu, 2017	Slovakia	☒	
*C. cameroni* Campbell and Pelham-Clinton, 1960	Great Britain	☒	☒
*C. cataneii* Clastrier, 1957	Algeria	☒	☒
*C. caucoliberensis* Callot, Kremer, Rioux and Descours 1967	France		☒
*C. chiopterus* (Meigen), 1830	Europe	☒	☒
*C. circumscriptus* Kieffer, 1918	Tunisia	☒	☒
*C. clastrieri* Callot, Kremer and Déduit, 1962	France	☒	☒
*C. clintoni* Boorman, 1984	Great Britain	☒	
*C. comosioculatus* Tokunaga, 1956	Japan	☒	
*C. corsicus* Kremer, Leberre and Beaucournu-Saguez, 1971	France	☒	☒
*C. cryptipulicaris* Talavera, Muñoz-Muñoz, Verdún and Pagès, 2017	Spain		
***C. delta*** Edwards, 1939 (=*C. lupicaris* Downes and Kettle, 1952 in Borkent 2022)	Great Britain		
*C. derisor* Callot and Kremer, 1965	France	☒	☒
*C. dewulfi* Goetghebuer, 1936	Belgium	☒	☒
*C. duddingstoni* Kettle and Lawson, 1955	Great Britain	☒	☒
*C. dzhafarovi* Remm, 1967	Azerbaijan	☒	
*C. fagineus* Edwards, 1939	Great Britain	☒	☒
*C. fascipennis* (Staeger), 1839	Denmark	☒	☒
*C. festivipennis* Kieffer, 1914	Germany	☒	☒
*C. flavipulicaris* Dzhafarov, 1964	Azerbaijan	☒	☒
*C. furcillatus* Callot, Kremer and Paradis, 1962	France	☒	☒
*C. gejgelensis* Dzhafarov, 1964	Azerbaijan	☒	☒
*C. griseidorsum* Kieffer, 1918	Algeria	☒	☒
*C. grisescens* Edwards, 1939	Great Britain	☒	
*C. haranti* Rioux, Descours and Pech, 1959	France	☒	☒
*C. heliophilus* Edwards, 1921	Great Britain	☒	
*C. helveticus* Callot, Kremer and Déduit, 1962	Switzerland	☒	
*C. heteroclitus* Kremer and Callot, 1965	France	☒	
*C. ibericus* Dzhafarov, 1963	Azerbaijan	☒	
*C. imicola* Kieffer, 1913	Kenya	☒	☒
*C. impunctatus* Goetghebuer, 1920	Belgium	☒	
*C. indistinctus* Khalaf, 1961	Iraq	☒	☒
*C. jamaicensis* Edwards, 1922 (*=C. paolae* Boorman 1996)	Jamaica		☒
*C. jumineri* Callot and Kremer, 1969	Tunisia	☒	☒
*C. jurensis* Callot, Kremer and Déduit, 1962	France	☒	
*C. kibunensis* Tokunaga, 1937	Japan	☒	☒
*C. kurensis* Dzhafarov, 1960	Azerbaijan	☒	☒
*C. longipennis* Khalaf, 1957	Iraq	☒	☒
***C. lupicaris*** Downes and Kettle, 1952 (= *C. delta* Edwards, 1939 in Borkent, 2022)	Great Britain	☒	☒
*C. malevillei* Kremer and Coluzzi, 1971	Italy	☒	☒
*C. manchuriensis* Tokunaga, 1941	China	☒	
*C. maritimus* Kieffer, 1924 (=*C. submaritimus* Dzhafarov, 1962 in Borkent, 2022)	Germany	☒	☒
***C. maritimus pauscisensillatus*** Callot, Kremer and Rioux, 1963	France	☒	
*C. minutissimus* (Zetterstedt), 1855	Sweden	☒	☒
*C. montanus* Shakirzjanova, 1962	Kazakhstan		☒
*C. newsteadi* Austen, 1921	Israel	☒	☒
*C. nubeculosus* (Meigen), 1830	Europe	☒	
*C. obsoletus* (Meigen), 1818	Europe	☒	☒
*C. odiatus* Austen, 1921	Israel	☒	☒
***C. pallidicornis/subfasciipennis* Kieffer, 1919**	Hungary	☒	☒
*C. paradisionensis* Boorman, 1988	Greece		☒
*C. paradoxalis* Ramilo and Delécolle, 2013	France	☒	☒
*C. parroti* Kieffer, 1922	Algeria	☒	☒
*C. pictipennis* (Staeger), 1839	Denmark	☒	☒
*C. picturatus* Kremer and Déduit, 1961	France	☒	☒
*C. poperinghensis* Goetghebuer, 1953	Belgium	☒	☒
*C. pseudopallidus* Khalaf, 1961	Iraq	☒	
*C. pulicaris* (Linnaeus), 1758	Europe	☒	☒
*C. punctatus* (Meigen), 1804	Europe	☒	☒
*C. puncticollis* (Becker), 1903	Egypt	☒	☒
*C. reconditus* Campbell and Pelham-Clinton, 1960	Great Britain	☒	
*C. riebi* Delécolle, Mathieu and Baldet, 2005	France	☒	☒
*C. riethi* Kieffer, 1914	Germany	☒	
*C. riouxi* Callot and Kremer, 1961	France	☒	☒
*C. saevus* Kieffer, 1922	Algeria	☒	
*C. sahariensis* Kieffer, 1923	Algeria	☒	
*C. salinarius* Kieffer, 1914	Germany	☒	☒
*C. santonicus* Callot, Kremer, Rault and Bach, 1966	France	☒	☒
*C. scoticus* Downes and Kettle, 1952	Great Britain	☒	☒
*C. segnis* Campbell and Pelham-Clinton, 1960	Great Britain	☒	
*C. semimaculatus* Clastrier, 1958	Algeria	☒	☒
*C. shaklawensis* Khalaf, 1957	Iraq	☒	☒
*C. simulator* Edwards, 1939	Great Britain	☒	
*C. sphagnumensis* Williams, 1955	USA (Michigan)	☒	
*C. stigma* (Meigen), 1818	Europe	☒	
*C. subfagineus* Delécolle and Ortega, 1998	Spain	☒	☒
***C. subfasciipennis/pallidicornis* Kieffer, 1919**	Hungary	☒	☒
***C. submaritimus*** Dzhafarov, 1962 (=*C. maritimus* Kieffer, 1924 in Borkent, 2022)	Azerbaijan	☒	☒
*C. tauricus* Gutsevich, 1959	Ukraine	☒	
*C. tbilisicus* Dzhafarov, 1964	Azerbaijan, Georgia	☒	☒
*C. truncorum* Edwards, 1939	Great Britain	☒	☒
*C. univittatus* Vimmer, 1932	Israel	☒	☒
*C. vexans* (Staeger), 1839	Denmark	☒	
*C. vidourlensis* Callot, Kremer, Mollet and Bach, 1968	France	☒	☒

The species name is based on the catalogue compiled by Borkent and Dominiak (1). In bold: discrepancies between the catalogue and Venail et al. (16).

**Table 2:** Cattle preference of *Culicoides* species in France based on molecular analysis of engorged *Culicoides* females and other techniques (animal baits, direct aspiration, light traps, glue strips).

Species	Indirect methods	Modern tools	Molecular and indirect studies
	References (2, 3, 4, 5, 6, 7, 8, 10, 11, 19, 24)	References (1, 8, 9, 12, 13, 14, 15, 16, 17, 18, 20, 21, 22, 23)	Reference (1)
*C. achrayi*	☒	☒	☒
*C. albicans*	☒		☒
*C. brunnicans*		☒	☒
*C. cataneii*	☒		☒
*C. chiopterus*	☒	☒	☒
*C. circumscriptus*	☒	☒	☒
*C. deltus*		☒	☒
*C. dewulfi*	☒	☒	☒
*C. fagineus*	☒ **¥**		☒
*C. fascipennis*	☒		☒
*C. festivipennis*	☒		☒
*C. furcillatus*		☒	☒
*C. gejgelensis*	☒		☒
*C. griseidorsum*	☒	☒	☒
*C. griescens*	☒	☒	☒
*C. haranti*	☒ **¥**		☒
*C. heliophilus*	☒		☒
*C. imicola*	☒	☒	☒
*C. impunctatus*	☒		☒
*C. jumineri*		☒	☒
*C. kibunensis*	☒	☒	☒
*C. longipennis*	☒		☒
*C. lupicaris*	☒	☒+L2	☒
*C. maritimus*	☒		☒
*C. minutissimus*	☒		
*C. montanus*	☒ **¥**		☒
*C. newsteadi*	☒	☒	☒
*C. obsoletus*	☒	☒	☒
*C. pallidicornis*	☒	☒	☒
*C. pictipennis*		☒	☒
*C. picturatus*		☒	☒
*C. poperinghensis*		☒	☒
*C. pulicaris*	☒	☒	☒
*C. punctatus*	☒	☒	☒
*C. puncticollis*	☒	☒	☒
*C. riethi*		☒	☒
*C. scoticus*	☒	☒	☒
*C. segnis*	☒	☒	☒
*C. semimaculatus*	☒ **¥**		☒
*C. shaklawensis*	☒		☒
*C. stigma*	☒		☒
*C. subfagineus*	☒		☒
*C. subfasciipennis*	☒		☒
*C. submaritimus*		☒	
*C. vexans*	☒	☒	☒
*C. obsoletus/scoticus*	☒	☒	
*C. subfascipennis/pallidicornis*		☒	
*C.* sp. *nr. Newsteadi*	☒		

¥: Unspecified. References (see Supplementary Table S2).

Secondly, documents, selected by Prudhomme et al. (19), are analysed in Supplementary Table S1. Publications selected (focus on biting midges) were reviewed in full text for inclusion of cattle (cattle OR livestock OR bovine OR cow OR beef OR calf OR calves OR heifer).

## Results

In all, we found 45 species that take blood meals from cattle (Table 2). Four species (*C. fagineus* Edwards *C. haranti* Rioux, Descours and Pech*, C. montanus* Shakirzjanova and *C. semimaculatus* Clastrier) have been reported to feed on cattle without information as to the method of collection (noted ¥ in Table 2). Finally, 14 species (*C. albicans* (Winnertz), *C. cataneii* Clastrier, *C. fascipennis* (Staeger), *C. festivipennis* Kieffer, *C. gejgelensis* Dzhafarov, *C. heliophilus* Edwards, *C. impunctatus* *C. longipennis* Khalaf, *C. maritimus* Kieffer, *C. minutissimus*, *C. shaklawensis* Khalaf, *C. stigma* (Meigen). *C. subfagineus* Delécolle and Ortega and *C. subfasciipennis* Kieffer) have been reported to feed on cattle based only on indirect investigations.

Second, based on 62 number of articles reviewed, only 4 articles, were used to compile the current species distribution list among the cattle (21–24). Overall, 18 species were reported in cattle: *C. achrayi* Kettle and Lawson*, C. brunnicans* Edwards, *C. chiopterus* (Meigen), *C. deltus* Edwards, *C. dewulfi* Goetghebuer, *C. duddingstoni* Kettle and Lawson, *C. festivipennis*, *C. furcillatus* Callot, Kremer and Paradis, *C. lupicaris* Downes and Kettle, *C. minutissimus*, *C. newsteadi* Austen*, C. obsoletus* (Meigen), *C. pallidicornis* Kieffer, *C. pictipennis* (Staeger), *C. pulicaris* (Linnaeus), *C. punctatus* (Meigen), *C. scoticus* Downes and Kettle, *C. vexans* Obsoletus group and *C. obsoletus/C. scoticus*.

## Discussion

The epidemiology of vector-borne diseases is linked to the preferred host and feeding behaviour of the arthropod vector. Our results reveal that 45 species feed on cattle (Table 2). The vertebrate hosts of 31 (9) and 37 (10) *Culicoides* species have previously been identified by molecular means. Indirect and molecular biology methods are in fact compatible (Table 2). There are more reports on the preferred hosts of *Culicoides* spp. by indirect methods than molecular tools (Table 2). Finally, research using proteomics to identify host proteins reveals a more diverse host pool than research using a traditional molecular approach (24). For *C. imicola* Kieffer, for example, the number of hosts varies between 1.5 and 5. Future studies on blood meal sources (i.e., hosts) could use a combination of PCR and proteomics-based methods to better characterise the *Culicoides* host spectrum.

Prudhomme et al. (19) proposed applying a step with three items (France OR Corsica OR French) AND (cattle OR livestock OR bovine OR cow OR beef OR calf OR calves OR heifer) AND (haematophag* OR hematophag* OR vector* OR arthropod* OR insect* OR tick* OR mite* OR acari*) to search for literature on the presence of *Culicoides* among cattle. They reported 94 *Culicoides* species recorded on different types of cattle farms in mainland France and Corsica. However, their review contains many mistakes: (i) many of the references are based on larval ecology (25–30) and these papers did not describe either the animals or any farms in the vicinity of the muds collected; (ii) references (25, 31–33) do not comply with the item “cattle OR livestock OR bovine OR cow OR beef OR calf OR calves OR heifer”; (iii) *C. cameroni* Campbell and Pelham-Clinton and *C. clintoni* Boorman were collected in natural areas and not on farms (34), so *C. clintoni* was not found with cattle; (iv) many *Culicoides* have been collected through the national surveillance programme in France (35). Traps were implemented among several types of livestock in both Corsica [sheep, cattle, horses (36); sheep (37); sheep, cattle (16)] and the French mainland [sheep (37); sheep and cattle (16, 21, 38, 39)]. Thus, the references using results of *Culicoides* trapped during national surveillance programme activities did not separate farms with cattle only from farms with other animals too. Consequently, the resulting list, which is said to focus only on cattle, is unreliable; (v) at the beginning of the *Culicoides* national surveillance programme, the composition of the Pulicaris group included three species (*C. pulicaris, C. lupicaris* Downes and *C. flavipulicaris* Dzhafarov) (16). Nowadays, in France the Pulicaris group includes six species (*C. boyi, C. bysta, C. cryptipulicaris*, *C. pulicaris, C. lupicaris* and *C. flavipulicaris*) (see Material and Methods and Table 1) but this new group has not been incorporated in the last publication (40); (vi) *Culicoides ibericus* Dzhafarov, *C. manchuriensis* Tokunaga*, C. sahariensis*, *C. tauricus* and *C. vexans* are found in mainland France but not Corsica (16); (vii) *C. pseudopallidus* Khalaf 1961 is present in France but *C. pseudoheliophilus* is not (16); (viii) finally, *C. accraensis* Carter, Ingram and Macfie, *C. albipennis* Kieffer*, C. musicola* (invalid species)*, C. pumilus* (Winnertz)*, C. sergenti* (Kieffer) and *C. sigrosignatus* (invalid species) have not been reported in France (14, 16). In our opinion, the *Culicoides* species presented in this systematic review (19) are not aligned with field reality. Our data show that 18 species are present among cattle in France (mainland without Corsica). The review of Prudhomme et al. (19) uses much results obtained by the national surveillance. But publications did not separate farms with cattle only from farms with other animals (See above). A great deal of vigilance must be exercised when we elaborate a review (based on requests in databases), in particular to choose of items.

The references used by Prudhomme et al. (19) include four techniques for characterising the presence of *Culicoides* biting midges among cattle: animal baits, larval ecology, light traps and the blood meals of *Culicoides* females. While light traps offer a good description of diversity (7), they do not accurately reflect the proportions of biting midges in an area (41). The light trap is not suited for midges feeding on an animal (42). Trapped engorged females with a blood meal in their abdomen can be used to accurately reveal host preferences by identifying the origin of the blood meal, but there is a flagrant bias because only night species are caught, and the attractiveness of light traps is limited. Animal baits can be used to identify host preferences and attack rates by counting and identifying the numbers of biting midges that can be captured from a specific host. Larval ecology offers knowledge of substrates suitable for *Culicoides* larval development. This method is not suitable for investigating relationships between a species and a host [in Prudhomme et al. (19) there is only one reference (23)]. A study on Belgian cattle farms has shown that 13 *Culicoides* species were obtained by incubation of soil samples (43) with 11 species presenting on cattle. In contrast, Uslu and Dik (44) describes the breeding sites of 18 *Culicoides* species in Turkey without describing the environmental sites. Future studies on larval development in the immediate surroundings of cattle farms will shed light on the microhabitats of *Culicoides* biting midges.

Five criteria are used to consider an arthropod as a biological vector of arboviruses (45, 46), including the presence of virus (es) and abundance of the suspected vector. Light traps underestimate the numbers of *Culicoides* present in an area (41), so *C. chiopterus* (Meigen) had not been seriously considered as a potential vector of BTV (41). The French national surveillance programme (35) has reported species in “groups or complex” to show species distribution and seasonal dynamics (16). Epidemiological studies can use wing characteristics to group together species with similar markings without needing to mount the specimens (47). But in this case, the results are not suitable for characterising the abundance of specific species. But, surveillance programme allows to model the abundance of *Culicoides* spp. in order to identify risk periods in France (48).

Contacts between competent vertebrate hosts and insect vectors are vital for vector-borne pathogens to successfully complete their transmission cycle (49). Cattle are very attractive to *Culicoides*, with 45 species feeding on them (Table 2) out of 92 species present in France. Among the species that feed on cattle, *C. chiopterus, C. dewulfi, C. imicola, C. obsoletus, C. pulicaris, C. punctatus, C. scoticus* are confirmed or probable vectors of BTV (50) and SBV (51). In addition, two species of the *newsteadi* complex and two species (*C. nubeculosus* *C. lupicaris*) are recorded or suspected of being involved in BTV and SBV transmission (50, 51), respectively. Most *Culicoides* species are opportunistic and may change host depending on availability (9, 10). For example, *C. scoticus* can switch from its preferential (predominant) mammal host to other hosts according to site and host availability (20). Many *Culicoides* species are known to feed on birds and to transmit the avian *Haemoproteus* parasite (52, 53). The ornithophilic *Culicoides* list includes 18 species (*C. alazanicus* Dzhafaraov, *C. cataneii, C. chiopterus, C. circumscriptus* Kieffer, *C. clastrieri* Callot, Kremer and Déduit, *C. festivipennis, C. griseidorsum* Kieffer, *C. impunctatus* *C. kibunensis* Tokunaga, *C. obsoletus, C. pallidicornis, C. pictipennis*, *C. pulicaris, C. punctatus, C. scoticus, C. segnis*, *C. seminaculatus* and *C. univittatus* Vimmer) (9, 52–54). Fourteen of these species may occasionally switch to cattle (Table 2). In contrast, several species usually take their blood meals from mammals or birds indifferently, and can switch from one to another (9, 10). The relationship between insects and their host is thus a key factor in the transmission of pathogens, including host availability (attractiveness and acceptability) (55). *Culicoides* species feed disproportionately on different host species (11, 42). For example, *Culicoides* spp. have been reported as feeding on cattle nine times more than on sheep (42), showing their attraction to cattle over sheep (11). Nevertheless, host diversity impacts *Culicoides* species diversity (7, 11, 22, 34, 42). *Culicoides* use hosts differently according to their preferential feeding locations on the body. Moreover, in one study *Culicoides* species switched between hosts (a cow and ewe) and between parts of the body attacked (42). More biting midges were collected from the head than the back, belly/flank and legs (11). One possible explanation could be the adaptation of *Culicoides* to a preferential (predominant) host through specific morphology of antennae, palpi and the number and/or distribution of sensilla (9, 10). This means that the sensory structures can be used to distinguish between ornithophilic, mammalophilic, or ornithophilic & mammalophilic species. *Culicoides* blood meals are not systematically included in *Culicoides* research literature (only 24 references in the Palaeartic region). As discussed, it is nonetheless a key factor for identifying the host spectrum of a species in order to anticipate/understand the transmission of pathogens. Hence, further studies that include both molecular approaches and indirect investigations and that focus on host preference by *Culicoides* species with nocturnal and diurnal activities would provide greater insights into *Culicoides* feeding patterns and vector-host interactions.

In conclusion, our study investigated the relationship between cattle and biting midges based on direct and indirect investigations. Data in the literature reports that all *Culicoides* species in France are present among cattle but our results reveal that only 18 species are present among cattle and 45 species feed on cattle out of the 92 species present. It could be misleading to state that species found near cattle interact with those cattle through blood meals, and research should focus on proving the midge vector-host relationship through both modern and traditional means in order to identify both the species involved in viral transmission and the viruses transmitted.

## Data Availability

The original contributions presented in the study are included in the article/Supplementary material, further inquiries can be directed to the corresponding authors.
