# Supplementary material for: Culicoides biting midges among cattle in France: be wary of data in the literature
Source: Front Vet Sci. 2024 Oct 24;11:1451442. doi: 10.3389/fvets.2024.1451442 (PMC11540827; doi:10.3389/fvets.2024.1451442)
Supplement: Supplementary file 1 [file Table_1.docx]

**References – Table 1**

1-Arnold, P. and M. Kremer (1982). Preliminary study for a mosquito eradication operation in Northern Bas-Rhin (France); Possible effects on Ceratopogonodae. Proceedings of the Fifth International Symposium on Ceratopogonidae, Strasbourg.

2-Augot, D., F. Sauvage, D. Jouet, E. Simphal, M. Veuille, A. Couloux, M. L. Kaltenbach and J. Depaquit (2010). "Discrimination of *Culicoides obsoletus* and *Culicoides scoticus*, potential bluetongue vectors, by morphometrical and mitochondrial cytochrome oxidase subunit I analysis." Infection, Genetics and Evolution **10**(5): 629-637.

3- Baldet, T., J. Delécolle, B. Mathieu, S. de La Rocque and F. Roger (2004). "Entomological surveillance of bluetongue in France in 2002." Veterinaria Italiana **40**(3): 226-231.

4- Baldet, T., B. Mathieu, J. Delecolle, G. Gerbier and F. Roger (2005). "Emergence de la fièvre catarrhale ovine dans le Bassin méditerranéen et surveillance entomologique en France." Revue d'Élevage et de Médecine Vétérinaire des Pays Tropicaux **58**(3): 125-132.

5- Baldet, T., J. C. Delecolle, C. Cêtre-Sossah, B. Mathieu, R. Meiswinkel and G. Gerbier (2008). "Indoor activity of *Culicoides* associated with livestock in the bluetongue virus (BTV) affected region of northern France during autumn 2006." Preventive Veterinary Medicine **87**(1-2): 84-97.

6- Balenghien, T., J.-C. Delécolle, M.-L. Setier-Rio, I. Rakotaoarivony, X. Allène, R. Venail, D. Delécolle, J. Lhoir, L. Gardès and D. Chavernac (2010). "Bluetongue-report on entomological surveillance in France in 2010." Bulletin Epidémiologique, Santé Animale et Alimentation **46**: 26-31.

7- Balenghien, T., J. C. Delecolle, M.-L. Setier-Rio, D. Delécolle, X. Allène, I. Rakotoarivony, B. Scheid, B. Mathieu, D. Chavernac and J.-B. Perrin (2013). "L'activité des populations de *Culicoides* en 2012 et bilan des quatre années du dispositif de surveillance." Bulletin Epidémiologique, Santé Animale et Alimentation **59**: 39-40.

8- Balenghien, T., I. Rakotoarivony, X. Allène, J.-B. Perrin and C. Garros (2014a). "L’activité des populations de *Culicoides* en Corse en 2013." Bulletin Epidémiologique, Santé Animale et Alimentation **64**: 45-46.

9- Balenghien, T., I. Rakotoarivony, X. Allène, J.-B. Perrin and C. Garros (2014b). "L’activité des populations de *Culicoides* en Corse en 2014." Bulletin Epidémiologique, Santé Animale et Alimentation **71**: 45-46.

10- Brumpt, E. (1942). "Notes parasitologiques concernant l’aménagement agricole de la Crau." Annales de Parasitologie Humaine et Comparée **19**(1-2-3): 74-84.

11- Cêtre-Sossah, C., T. Baldet, J.-C. Delécolle, B. Mathieu, A. Perrin, C. Grillet and E. Albina (2004). "Molecular detection of *Culicoides* spp. and *Culicoides imicola*, the principal vector of bluetongue (BT) and African horse sickness (AHS) in Africa and Europe." Veterinary Research **35**(3): 325-337.

12- Cetre-Sossah, C. (2010). Des parasites humains aux virus animaux, une histoire de vecteurs. HDR, Université de Montpellier 2.

13- Chacker, E. (1982). Description of larvae of six species of *Culicoides*. Proceedings of the Fifth International Symposium on Ceratopogonidae, Strasbourg.

14- Cuéllar, A. C., L. J. Kjær, C. Kirkeby, H. Skovgard, S. A. Nielsen, A. Stockmarr, G. Andersson, A. Lindstrom, J. Chirico and R. Lühken (2018). "Spatial and temporal variation in the abundance of *Culicoides* biting midges (Diptera: Ceratopogonidae) in nine European countries." Parasites and Vectors **11**(1): 1-18.

15- Delécolle, J.-C. and S. d. La Rocque (2002). "Contribution à l'étude des *Culicoides* de Corse. Liste des espèces recensées en 2000/2001 et redescription du principal vecteur de la Fièvre Catarrhale Ovine : *Culicoides imicola* Kieffer, 1913 (Diptera, Ceratopogonidae)." Bulletin de la Société Entomologique de France **107**(4): 371-379.

16- ECDC. (2022a). "European Centre for Disease Prevention and Control. Biting midge maps." Access date 10/01/2023, Available from <https://www.ecdc.europa.eu/en/disease-vectors/surveillance-and-disease-data/biting-midge-maps>.

17- Gauchard, F. and A.-M. Hattenberger (2005). Rapport sur l'évaluation du risque d'apparition et de développement de maladies animales compte tenu d'un éventuel réchauffement climatique. Paris (France), AFSSA

18- Garros, C., L. Gardes, X. Allene, I. Rakotoarivony, E. Viennet, S. Rossi and T. Balenghien (2011). "Adaptation of a species-specific multiplex PCR assay for the identification of blood meal source in *Culicoides* (Ceratopogonidae: Diptera): applications on Palaearctic biting midge species, vectors of Orbiviruses." Infection, Genetics and Evolution **11**(5): 1103-1110.

19- Garros, C. (2022). Complete data of *Culicoides* captures realized by the surveillance network in France in 2010.

20- GBIF. (2023). "Global Biodiversity Information Facility." Access date 20/02/2023, Available from <https://www.gbif.org/fr/>

21- Gerbier, G., F. Biteau‐Coroller, C. Grillet, J. Parodi, S. Zientara, T. Baldet, H. Guis and F. Roger (2008). "Description of the outbreak of Bluetongue in Corsica in 2003, and lessons for surveillance." Veterinary Record **162**(6): 173-176.

22- Gerbier, G., J. Parodi, F. Biteau-Coroller, T. Baldet, B. Mathieu, S. Zientara, C. Cêtre-Sossah and F. Roger (2006). "Surveillance de la Fiévre Catarrhale Ovine (Bluetongue) en France et dans l'ouest méditerranéen : Bilan et perspectives." Epidémiologie et Santé Animale **49**: 37-44

23- INPN. (2023). "Inventaire National du Patrimoine Naturel. Synthèse de données pour les espèces." Access date 20/02/2023, Available from <https://inpn.mnhn.fr/espece/indicateur/FR/ES/7/CL/PH/Arthropoda>.

24- Hajd Henni, L., F. Sauvage, C. Ninio, J. Depaquit and D. Augot (2014). "Wing geometry as a tool for discrimination of Obsoletus group (Diptera: Ceratopogonidae: *Culicoides*) in France." Infection, Genetics and Evolution **21**: 110-117.

25- Hendrikx, P. (2003). "Adaptation des réseaux de surveillance épidémiologique aux conditions de l'émergence." Epidémiologie et Santé Animale **44**: 51-59.

26- Jacquet, S., C. Garros, E. Lombaert, C. Walton, J. Restrepo, X. Allene, T. Baldet, C. Cetre‐Sossah, A. Chaskopoulou and J. C. Delecolle (2015). "Colonization of the Mediterranean basin by the vector biting midge species *Culicoides imicola*: an old story." Molecular Ecology **24**(22): 5707-5725

27- Jacquet, S., K. Huber, H. Guis, M.-L. Setier-Rio, M. Goffredo, X. Allène, I. Rakotoarivony, C. Chevillon, J. Bouyer and T. Baldet (2016a). "Spatio-temporal genetic variation of the biting midge vector species *Culicoides imicola* (Ceratopogonidae) Kieffer in France." Parasites and Vectors **9**(1): 1-12.

28- Jacquet, S., K. Huber, S. Talavera, L. E. Burgin, S. Carpenter, C. Sanders, A. H. Dicko, M. Djerbal, M. Goffredo and Y. Lhor (2016b). "Range expansion of the Bluetongue vector, *Culicoides imicola*, in continental France likely due to rare wind-transport events." Scientific Reports **6**(1): 1-1

29- Kieffer, J.-J. (1925). Diptères (Nématocères piqueurs) : Chironomidae Ceratopogoninae. Paris (France), Lechevalier P.

30- Kluiters, G., S. Carpenter, L. Gardes, H. Guis, M. Baylis and C. Garros (2016). "Morphometric discrimination of two sympatric sibling species in the Palaearctic region, *Culicoides obsoletus* Meigen and *C. scoticus* Downes & Kettle (Diptera: Ceratopogonidae), vectors of Bluetongue and Schmallenberg viruses." Parasites and Vectors **9**(1): 1-15.

31- Kremer, M. (1965). Contribution à l'étude du genre *Culicoides* Latreille, particulièrement en France. Paris (France), Lechevalier P.

32- Kremer, M., G. Leberre and F. Beaucournu-Saguez (1971). "Notes sur les *Culicoides* (Dipt. Ceratopogonidae) de Corse. Description de *C. corsicus* n. sp." Annales de Parasitologie Humaine et Comparée **46**(5): 653-660.

33- Kremer, M., J. Rieb and C. Rebholtz (1978). "[Ecology of the Ceratopogonids of the Alsace plain. I. The genus *Culicoides* from the humid soils of the Ried]." Annales de Parasitologie Humaine et Comparée **53**(1): 101-115.

34- Mathieu, B. (2011). Les espèces de *Culicoides* du sous-genre *Avaritia* (Diptera : Ceratopogonidae) dans le monde: révision systématique et taxonomique des espèces d'intérêt dans la transmission d'*Orbivirus*, Université de Strasbourg.

35- Mathieu, B., J.-C. Delecolle, C. Garros, T. Balenghien, M.-L. Setier-Rio, E. Candolfi and C. Cêtre-Sossah (2011). "Simultaneous quantification of the relative abundance of species complex members: application to *Culicoides obsoletus* and *Culicoides scoticus* (Diptera: Ceratopogonidae), potential vectors of bluetongue virus." Veterinary Parasitology **182**(2-4): 297-306.

36- Mehlhorn, H. (2012). Arthropods as vectors of emerging diseases. Londres (Angleterre), Springer Science & Business Media.

37- Meiswinkel, R., T. Baldet, R. De Deken, W. Takken, J. Delécolle and P. Mellor (2007). Epidemiological analysis of the 2006 blue tongue virus serotype 8 epidemic in North-Western Europe. Distribution and dynamics of vector species**:** 88.

38- Mellor, P. (2004). "Infection of the vectors and Bluetongue epidemiology in Europe." Veterinaria Italiana **40**(3): 176-181.

39- Mellor, P. S. and E. J. Wittmann (2002). "Bluetongue virus in the Mediterranean basin 1998–2001." Veterinary journal **164**(1): 20-37.

40- Mialhe, E., C. Louis, J.-P. Quiot, J.-P. Rieb and C. Vaga (1982). Evidence and study of a Chlamydial infection in *Culicoides* sp. Proceedings of the Fifth International Symposium on Ceratopogonidae, Strasbourg.

41- Mignotte, A. (2020). Maladies émergentes en santé animale: diversité génétique et dispersion d'une espèce de moucheron vectrice des virus de la Fièvre Catarrhale Ovine et de Schmallenberg en région paléarctique, *Culicoides obsoletus* (Diptera: Ceratopogonidae), Université de Montpellier.

42- Mignotte, A., C. Garros, S. Dellicour, M. Jacquot, M. Gilbert, L. Gardès, T. Balenghien, M. Duhayon, I. Rakotoarivony and M. de Wavrechin (2021). "High dispersal capacity of *Culicoides obsoletus* (Diptera: Ceratopogonidae), vector of bluetongue and Schmallenberg viruses, revealed by landscape genetic analyses." Parasites and Vectors **14**(1): 1-14.

43- Mignotte, A., C. Garros, L. Gardès, T. Balenghien, M. Duhayon, I. Rakotoarivony, L. Tabourin, L. Poujol, B. Mathieu and A. Ibañez-Justicia (2020). "The tree that hides the forest: cryptic diversity and phylogenetic relationships in the Palaearctic vector Obsoletus/Scoticus Complex (Diptera: Ceratopogonidae) at the European level." Parasites and Vectors **13**(1): 1-13.

44- Ninio, C. (2011). Fièvre Catarrhale Ovine dans les Ardennes : étude de la biologie des *Culicoides* et de leur rôle épidémiologique, Reims.

45- Ninio, C., D. Augot, J. C. Delecolle, B. Dufour and J. Depaquit (2011a). "Contribution to the knowledge of *Culicoides* (Diptera: Ceratopogonidae) host preferences in France." Parasitology Research **108**(3): 657-663.

46- Ninio, C., D. Augot, B. Dufour and J. Depaquit (2011b). "Emergence of *Culicoides obsoletus* from indoor and outdoor breeding sites." Veterinary Parasitology **183**(1-2): 125-129.

47- OIE (2000). "Bluetongue in France in the island of Corsica." OIE news **13**(43): 195-197.

48- Perrin, A., C. Cetre‐Sossah, B. Mathieu, T. Baldet, J. C. Delecolle and E. Albina (2006). "Phylogenetic analysis of *Culicoides* species from France based on nuclear ITS1‐rDNA sequences." Medical and Veterinary Entomology **20**(2): 219-228.

49- Rageau, J. and J. Mouchet (1967). "Les arthropodes hématophages de Camargue." Cahier ORSTOM: Série Entomologie Médicale et Parasitologie **5**(4): 263-281.

50- Ramilo, D., C. Garros, B. Mathieu, C. Benedet, X. Allene, E. Silva, G. Alexandre-Pires, I. P. Da Fonseca, S. Carpenter and J. Radrova (2013). "Description of *Culicoides paradoxalis* sp. nov. from France and Portugal (Diptera: Ceratopogonidae)." Zootaxa **3745**(2): 243-256.

51- Rieb, J.-P. (1987). "L'estivo-hibernation et le contrôle de la dynamique du cycle évolutif dans le genre *Culicoides* (Diptères, Cératopogonidés)." Vie et Milieu **37**(1): 23-37.

52- Rioux, J.-A., S. Descous and J. Pech (1959). "Un nouveau Cératopogonide arboricole : *Culicoides haranti* n. sp. (Diptera, Heleidae)." Annales de Parasitologie Humaine et Comparée **34**(3): 432-438.

53- Rossi, S., T. Balenghien, C. Viarouge, E. Faure, G. Zanella, C. Sailleau, B. Mathieu, J.-C. Delécolle, C. Ninio, C. Garros, L. Gardès, C. Tholoniat, A. Ariston, D. Gauthier, S. Mondoloni, A. Barboiron, M. Pellerin, P. Gibert, C. Novella, S. Barbier, E. Guillaumat, S. Zientara, D. Vitour and E. Bréard (2019). "Red deer (*Cervus elaphus*) did not play the role of maintenance host for Bluetongue virus in France: the burden of proof by long-term wildlife monitoring and *Culicoides* snapshots." Viruses **11**(10): 903-929.

54- Sailleau, C., E. Bréard, G. Gerbier, J. Parodil, A. Bouchot and S. Zinetara (2005). "Épidémiologie descriptive et moléculaire de la Bluetongue en Corse en 2004." Epidémiologie et Santé Animale **48**: 9-14.

55- Tran, A., F. Biteau-Coroller, H. Guis and F. Roger (2005). "Modélisation des maladies vectorielles." Epidémiologie et Santé Animale **47**: 35-51

56- Venail, R. (2014). Sensibilité aux insecticides et évaluation préliminaire des méthodes de lutte antivectorielle disponibles contre les *Culicoides* (Diptera : Ceratopogonidae) Paléarctiques, vecteurs de virus émergents d'intérêt en santé animale, Université de Montpellier 2.

57- Viennet, E. (2011). Insectes et maladies émergentes : Contacts hôte/*Culicoides* en région Paléarctique et leurs implications dans la transmission de la Fièvre Catarrhale Ovine, Université de Montpellier 2.

58- Viennet, E., C. Garros, I. Rakotoarivony, X. Allene, L. Gardès, J. Lhoir, I. Fuentes, R. Venail, D. Crochet and R. Lancelot (2012). "Host-seeking activity of Bluetongue virus vectors: endo/exophagy and circadian rhythm of *Culicoides* in Western Europe." PLOS One **7**(10): e48120.

59- Viennet, E., C. Garros, L. Gardes, I. Rakotoarivony, X. Allene, R. Lancelot, D. Crochet, C. Moulia, T. Baldet and T. Balenghien (2013). "Host preferences of Palaearctic *Culicoides* biting midges: implications for transmission of orbiviruses." Medical and Veterinary Entomology **27**(3): 255-266.

60- Waller, J., M. Kremer and D. J.C. (1982). Turf-moss *Culicoides* in the Hautes-Vosges (France). Proceedings of the Fifth International Symposium on Ceratopogonidae, Strasbourg.

61- Zientara, S., S. De La Rocque, J.-M. Gourreau, M. Grégory, A. Diallo, P. Hendrikx, G. Libeau, C. Sailleau and J. C. Delecolle (2000). "La Fièvre Catarrhale Ovine en Corse en 2000." Epidémiologie et Santé Animale **38**: 133-144.

62- Zientara, S., C. Grillet, S. De la Rocque, J. M. Gourreau, M. Grégory, P. Hendrikx, G. Libeau, C. Sailleau, E. Albina, E. Bréard and J. C. Delécolle (2001). "La Fièvre Catarrhale Ovine en Corse en 2001." Epidémiologie et Santé Animale **40**: 129-134.
