# Supplementary material for: Culicoides biting midges among cattle in France: be wary of data in the literature
Source: Front Vet Sci. 2024 Oct 24;11:1451442. doi: 10.3389/fvets.2024.1451442 (PMC11540827; doi:10.3389/fvets.2024.1451442)
Supplement: Supplementary file 2 [file Table_2.docx]

**References – Table 2**

1. Augot D, Hadj-Henni L, Strutz S, Slama D, Millot C. Depaquit J, et al. Association between host species choice and morphological characters of main sensory structures of *Culicoides* in the Palaeartic region. *PeerJ* (2017) 5:e3478. doi: 10.7717/peerj.3478

2. Ayllón T, Nijhof AM, Weiher W, Bauer B, Allène X, Clausen PH. Feeding behaviour of *Culicoides spp*. (Diptera: Ceratopogonidae) on cattle and sheep in northeast Germany. *Parasite Vectors.* (2014) 7: 34. doi: 10.1186/1756-3305-7-34.

3. Braverman Y, Boreham PF, Galum R. The origin of bloodmeals of female *Culicoides pallidipennis* trapped in a sheepfold in Israel. *J. Med. Entomol.* (1971) 8: 379-381. doi: 10.1093/jmedent/8.4.379.

4. Braverman Y, Delecolle JC, Frish K, Rubina M, Kremer M. New records of *Culicoides* species (Diptera: Ceratopogonidae) from Golan Heights, Israel and Sinai Peninsula. *Israel J. Ent.* (1981) 15: l3-20.

5. Braverman Y, Linley JR. Fecundity and proportions of gravid females in populations the bluetongue vector *Culicoides imicola* (Diptera: Ceratopogonidae) and several other species in Israel. *J. Med. Entomol.* (1994) 3l, 838-843. doi: 10.1093/jmedent/31.6.838.

6. Braverman Y, Messadegg N, Lemble C, Kremer M. Reevaluation of the taxonomic status of the *Culicoides* spp (Diptera: Ceratopogonidae) from Israel and the Eastern Meditenanean and reviewed of their potential medical and veterinary importance. *J Amer Mosq Centr Assoc* (1996) 12:437445.

7.Braverman Y, Rechtman S, Frish A, Braverman R. Dynamics of biting avtivity of *C. imicola* Kieffer (Diptera: Ceratopogonidae) during the year. *Israel J. Vet. Med.* (2003) 58, 46-56.

8. Braverman Y, Frish K, Reis M, Mumcuoglu KY. Host preference of *Culicoides* spp. from Israel based on sensory organs and morphometry (Diptera: Ceratopogonidae).*Entomologica Generalis* (2012) 34:97-110.

9. Díaz-Sánchez S, Hernández-Jarguín A, Torina A, Fernández de Mera IG, Estrada-Peña A, Villar M, et al. [Biotic and abiotic factors shape the microbiota of wild-caught populations of the arbovirus vector *Culicoides imicola*.](https://pubmed.ncbi.nlm.nih.gov/30058755/) *Insect Mol. Bio.* (2018) 27(6): 847-861. doi: 10.1111/imb.12526.

10. Elbers ARW, Meiswinkel R. *Culicoides* (Diptera: Ceratopogonidae) host preferences and biting rates in the Netherlands: comparing cattle, sheep and the black-light trap. *Vet. Parasitol* (2014) 205: 330-337. doi: 10.1016/j.vetpar.2014.06.004.

11. Elbers ARW, Meiswinkel R. *Culicoides* (Diptera: Ceratopogonidae) and livestock in the Netherlands: comparing host preference and attack rates on a Shetland pony, a dairy cow, and a sheep. *J. Vect. Ecol.* (2015) 40(2): 308-317. doi: 10.1111/jvec.12169.

12. González MA, Goiri F, Prosser SWJ, Cevidanes A, Hernández-Triana LM, Barandika JF, et al. [*Culicoides* species community composition and feeding preferences in two aquatic ecosystems in northern Spain.](https://pubmed.ncbi.nlm.nih.gov/35690834/) *Parasit Vectors* (2022) 15(1): 199. doi: 10.1186/s13071-022-05297-5.

13. Kasičová Z, Schreiberová A, Kimáková A, Kočišová A. [Blood meal analysis: host-feeding patterns of biting midges (Diptera, Ceratopogonidae, *Culicoides* Latreille) in Slovakia.](https://pubmed.ncbi.nlm.nih.gov/34283022/) *Parasite* (2021) 28:58. doi: 10.1051/parasite/2021058.

14. Lassen SB, Nielsen SA, Skovgard H, Kristensen M. Molecular identification of bloodmeals from biting midges (Diptera: Ceratopogonidae: *Culicoides* Latreille) in Denmark. *Parasitol. Res*. (2011) 108(4): 823-829. doi: 10.1007/s00436-010-2123-4

15. Lassen SB, Nielsen S, Kristensen M. Identity and diversity of blood meal hosts of biting midges (Diptera: Ceratopogonidae: *Culicoides* Latreille) in Denmark. *Parasit Vectors* (2012) 5: 143. doi: 10.1186/1756-3305-5-143.

16. Martínez-de la Puente J, Figuerola J, Soriguer R. 2015. Fur or feather? Feeding preferences of species of *Culicoides* biting midges in Europe. *Trends Parasitol.* (2015) 31(1): 16-22. doi: 10.1016/j.pt.2014.11.002.

17. Ninio C, Augot D, Delecolle JC, Dufour B, Depaquit J. Contribution to the knowledge of *Culicoides* (Diptera: Ceratopogonidae) host preferences in France. *Parasitol. Res.* (2011) 108(3): 657-663. doi: 10.1007/s00436-010-2110-9.

18. Rossi S, Balenghien T, Viarouge C, Faure E, Zanella G, Sailleau C, et al. Red deer (*Cervus elaphus*) did not play the role of maintenance host for Bluetongue virus in France: the burden of proof by long-term wildlife monitoring and *Culicoides* snapshots*. Viruses* (2019) 11(10): 903–929. doi: 10.3390/v11100903.

19. Santiago-Alarcon D, Havelka P, Schaefer HM, Segelbacher G. Bloodmeal analysis reveals avian Plasmodium infections and broad host preferences of *Culicoides* (Diptera: Ceratopogonidae) vectors. *Plos One* (2012) 7(2): e31098. doi: 10.1371/journal.pone.0031098

20. Santiago-Alarcon D, Havelka P, Pineda E, Segelbacher G, Schaefer HM. Urban forests as hubs for novel zoonosis: blood meal analysis, seasonal variation in *Culicoides* (Diptera: Ceratopogonidae) vectors, and avian haemosporidians. *Parasitology* (2013) 140(14): 1799-1810. doi: 10.1017/S0031182013001285

21. Slama D, Haouas N, Mezhoud H, Babba H, Chaker E. Bloodmeal analysis of *Culicoides* (Diptera:Ceratopogonidae) in Central Tunisia. *PlosOne* (2015) 10(3), e0120528. doi: 10.1371/journal.pone.0120528.

22. Talavera S, Muñoz-Muñoz F, Verdún M, Pujol N, Pagès N. [Revealing potential bridge vectors for BTV and SBV: a study on *Culicoides* blood feeding preferences in natural ecosystems in Spain.](https://pubmed.ncbi.nlm.nih.gov/28857265/) *Med. Vet. Entomol.* (2018) 32(1): 35-40. doi: 10.1111/mve.12263.

23. Tomazatos A, Jöst H, Schulze J, Spînu M, Schmidt-Chanasit J, Cadar D, et al. [Blood-meal analysis of *Culicoides* (Diptera: Ceratopogonidae) reveals a broad host range and new species records for Romania.](https://pubmed.ncbi.nlm.nih.gov/32066493/) Parasite Vectors (2020) 13(1): 79. doi: 10.1186/s13071-020-3938-1.

24.Viennet E, Garros C, Gardes L, Rakotoarivony I, Allene X, Lancelot R, et al. Host preferences of Palaearctic *Culicoides* biting midges: implications for transmission of orbiviruses. *Med. Vet. Entomol.* (2013) 27(3): 255-266. doi: 10.1111/j.1365-2915.2012.01042.x.
